# Supplementary material for: Peroxisome biogenesis deficiency attenuates the BDNF-TrkB pathway-mediated development of the cerebellum
Source: Life Sci Alliance. 2018 Dec 3;1(6):e201800062. doi: 10.26508/lsa.201800062 (PMC6277683; doi:10.26508/lsa.201800062)

Full unedited gel for Figure 2 E

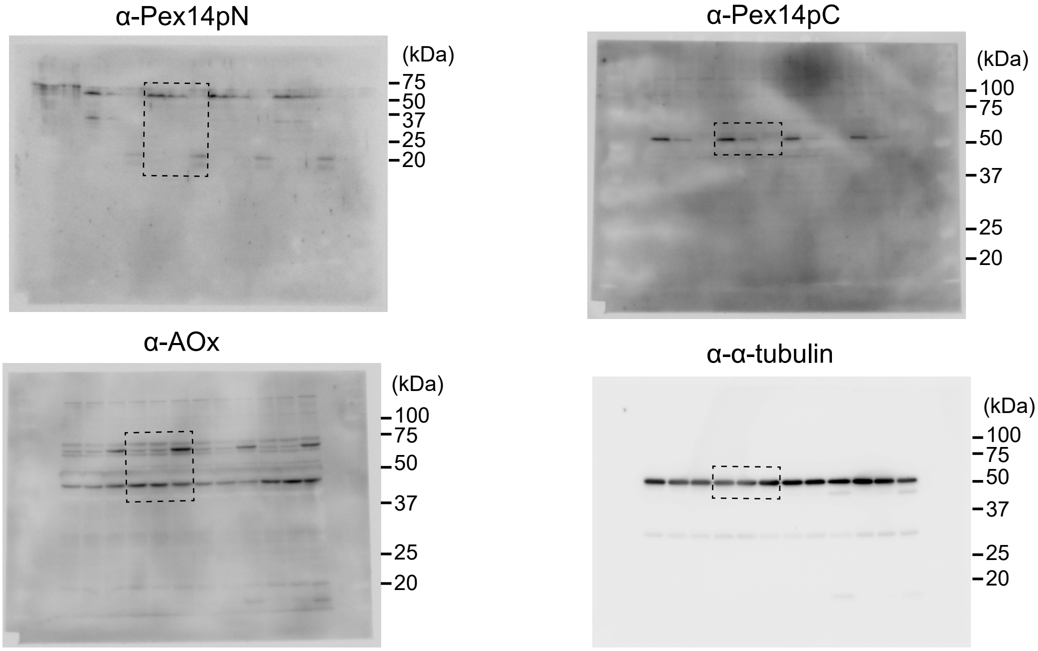

Full unedited gels for FIgure 4 E

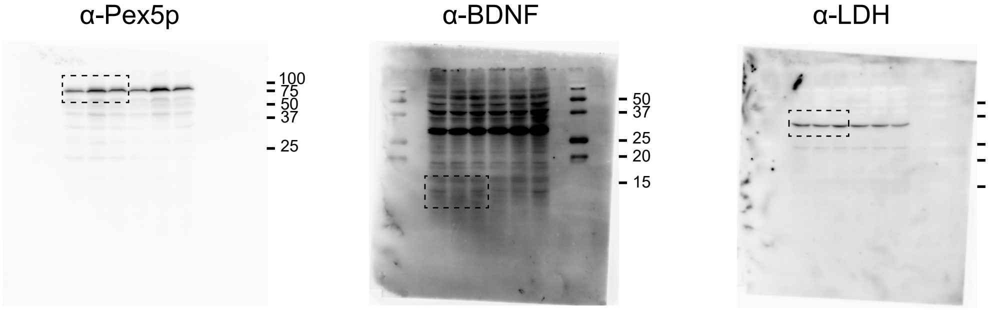

Full unedited gel for Figure 6 E

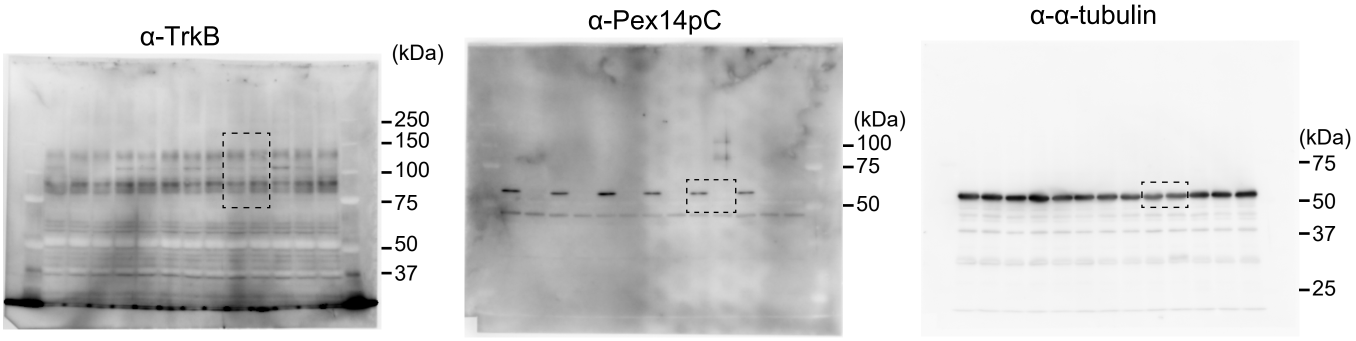

Full unedited gel for Figure 7 A

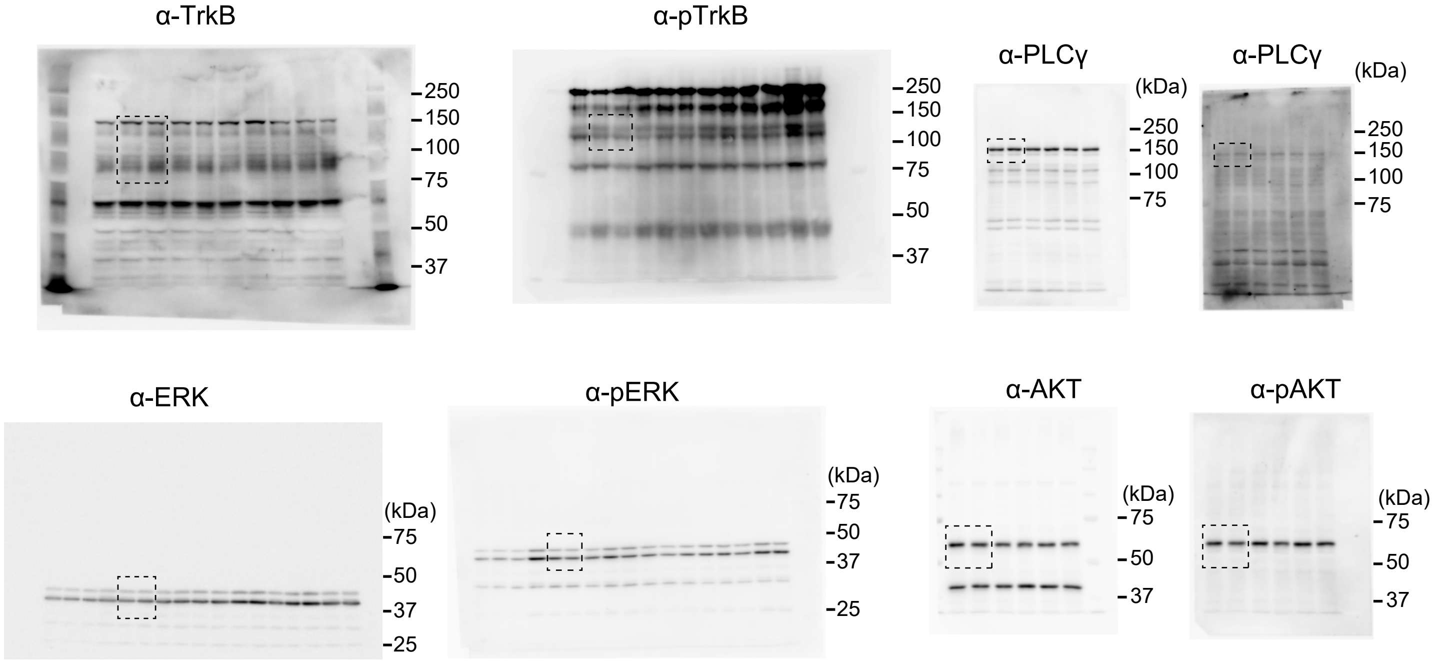

Full unedited gel for Figure 8 H

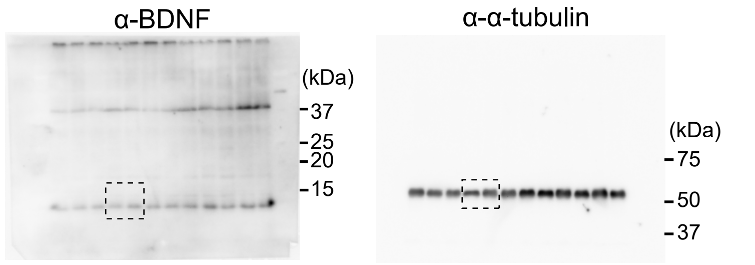

Full unedited gels for FIgure S1 A

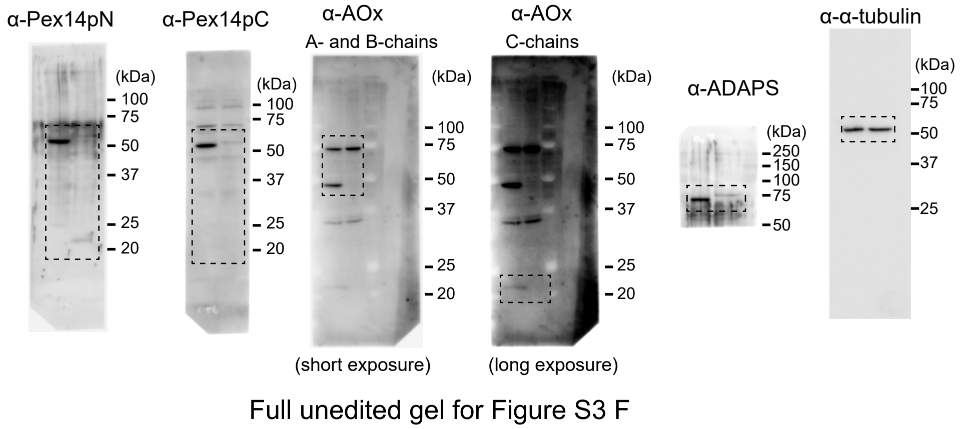

Full unedited gel for FIgure S3 F

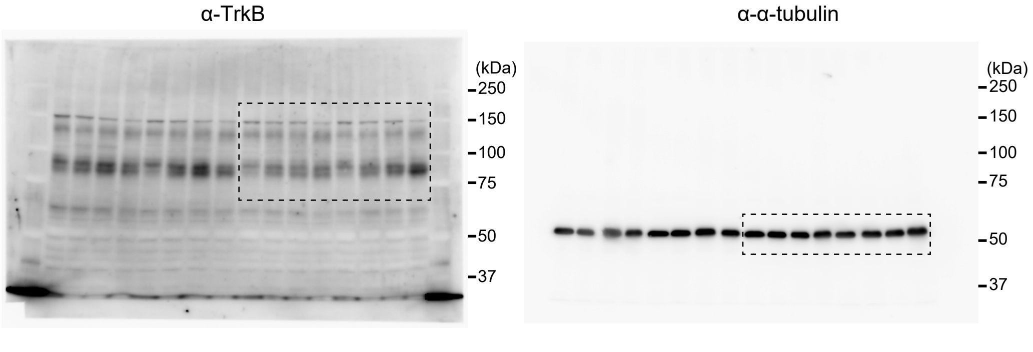

Full unedited gel for Figure S3 H

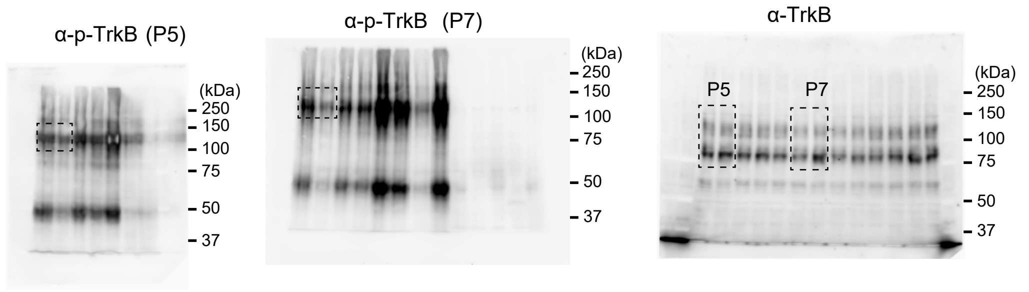

Full unedited gel for Figure S3 I

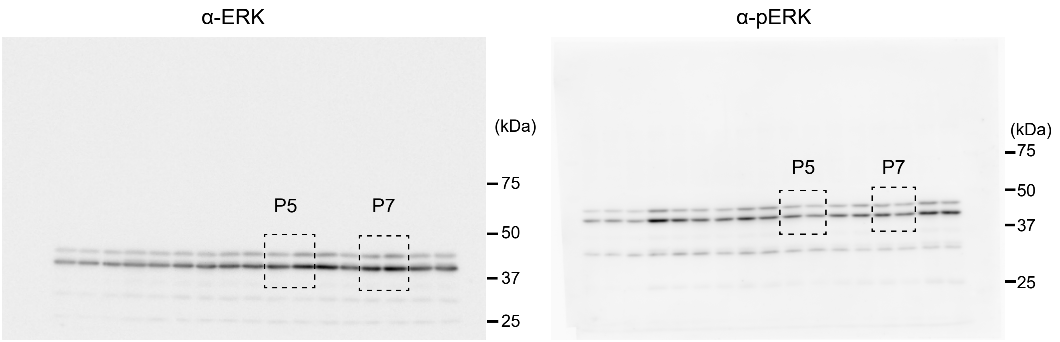

Full unedited gel for Figure S4 A

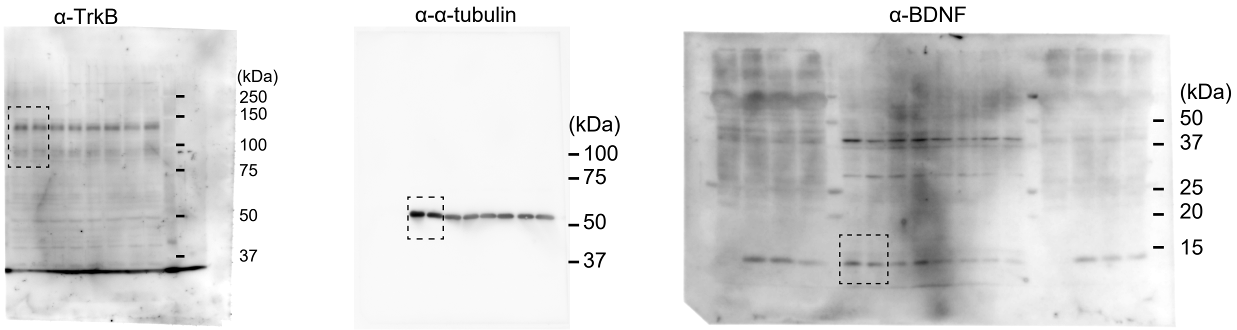

Full unedited gel for Figure S4 D

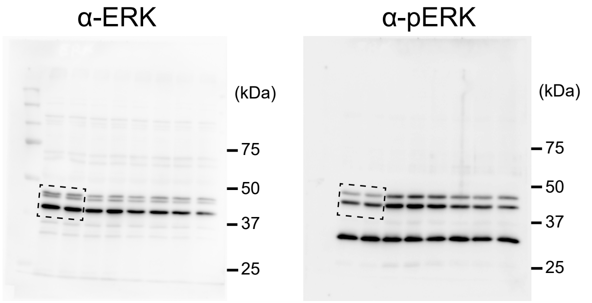

Full unedited gel for Figure S4 E

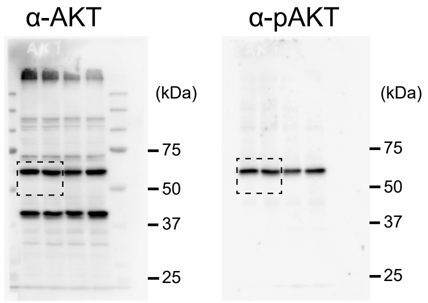

Supplement: Supplementary file 1 [file LSA-2018-00062_SdataF1.pdf]
